# An Exploratory Study on ^99m^Tc-RGD-BBN Peptide Scintimammography in the Assessment of Breast Malignant Lesions Compared to ^99m^Tc-3P4-RGD2

**DOI:** 10.1371/journal.pone.0123401

**Published:** 2015-04-07

**Authors:** Qianqian Chen, Qingjie Ma, Minglong Chen, Bin Chen, Qiang Wen, Bing Jia, Fan Wang, Butong Sun, Shi Gao

**Affiliations:** 1 Department of Nuclear Medicine, China-Japan Union Hospital of Jilin University, Changchun, 130033, China; 2 Medical Isotopes Research Center of Peking University, Beijing, 100191, China; Van Andel Institute, UNITED STATES

## Abstract

**Purpose:**

This study aimed to explore the diagnostic performance of single photon emission computed tomography / computerized tomography (SPECT/CT) using a new radiotracer ^99m^Tc-RGD-BBN for breast malignant tumor compared with ^99m^Tc-3P4-RGD_2_.

**Methods:**

6 female patients with breast malignant tumors diagnosed by fine needle aspiration cytology biopsy (FNAB) who were scheduled to undergo surgery were included in the study. ^99m^Tc-3P4-RGD_2_ and ^99m^Tc-RGD-BBN were performed with single photon emission computed tomography (SPECT) at 1 hour after intravenous injection of 299 ± 30 MBq and 293 ± 32 MBq of radiotracers respectively at separate day. The results were evaluated by the Tumor to non-Tumor ratios (T/NT). ^99m^Tc-RGD-BBN and ^99m^Tc-3P4-RGD_2_ SPECT/CT images were interpreted independently by 3 experienced nuclear medicine physicians using a 3-point scale system. All of the samples were analyzed immunohistochemically to evaluate the integrin αvβ3 and gastrin-releasing peptide receptor (GRPR) expression. The safety, biodistribution and radiation dosimetry of ^99m^Tc-RGD-BBN were also evaluated in the healthy volunteers.

**Results:**

No serious adverse events were reported in any of the patients during the study. The effective radiation dose entirely conformed to the relevant standards. A total of 6 palpable malignant lesions were detected using ^99m^Tc-RGD-BBN SPECT/CT with clear uptake. All malignant lesions were also detected using ^99m^Tc-3P4-RGD_2_ SPECT/CT. The results showed that five malignant lesions were with clear uptake and the other one with barely an uptake. 4 malignant cases were found with both αvβ3 and GRPR expression, 1 case with only GRPR positive expression (integrin αvβ3 negative) and 1 case with only integrin αvβ3 positive expression (GRPR negative).

**Conclusion:**

^99m^Tc-RGD-BBN is a safe agent for detecting breast cancer. ^99m^Tc-RGD-BBN may have the potential to make up for the deficiency of ^99m^Tc-3P4-RGD_2_ in the detection of breast cancer with only GRPR positive expression (integrin α_v_β_3_ negative). The preliminary application of ^99m^Tc-RGD-BBN has demonstrated its powerful potential in breast cancer diagnosis and therapy.

## Introduction

Breast cancer is the most frequent malignancy in women all over the world. In the United States, a Cancer Journal for Clinicians estimates that 234,580 women will be diagnosed with breast cancer in 2013 and expected to account for 30% of all female new cancers. Also, the incidence rate is increasing year by year in China [1,2]. Early detection of breast cancer may lead to a higher rate of successful treatment and extend patients’ lives.

X-ray mammography (XMM) and ultrasound (US) are now employed as conventional tools for breast tumor screening. The wide use of them in early detection of tumor has saved thousands of lives. With the advent of molecular imaging era,nuclear medicine techniques is considered promising in early detection of tumor through a functional perspective. Of them, scintimammography (SMM) with various targeted probes have become a major interest in this area [3]. Since the power of tracer largely decides the performance of a SMM examination, huge efforts have been paid in developing them. [^99m^Tc (HYNIC-3PRGD_2_) (tricine) (TPPTS)] (^99m^Tc-3P4-RGD_2_), a peptide with high binding affinity to integrin α_v_β_3_ on tumor cells, is a newly designed tracer and has shown remarkable performance in detecting tumor [4–7].

Expression of cell-surface receptors by cancer cells can be heterogeneous and inhomogeneous. Breast cancer cells, for example, over-express two receptors [gastrin-releasing peptide receptor (GRPR) in 70% breast cancer cells and integrin α_v_β_3_ in 58% breast cancer cells]. It is difficult to identify all breast cancer cells with just one target-based cancer imaging. Therefore, it is desirable to develop a new type of radiotracers that can target not just one but several different receptors simultaneously. Of course, the receptors need to be more specific peptide vs integrin receptors.

Recently, our laboratory designed and synthesized a dual integrin α_v_β_3_ and GRPR targeted peptide Glu-c(RGDyK)-bombesin (RGD-BBN) that contained dual RGD and BBN motifs in one molecule [8,9]. In an earlier study, the ^99m^Tc labeled RGD-BBN heterodimer exhibited excellent pharmacokinetics and biology distribution in the mouse models [9]. This study was designed to determine the safety, pharmacokinetics and biodistribution characteristics of ^99m^Tc-RGD-BBN in healthy volunteers for the first time. Furthermore, we aimed to explore the diagnostic performance of ^99m^Tc-RGD-BBN single photon emission computed tomography / computerized tomography (SPECT/CT) for palpable breast abnormalities, and to compare ^99m^Tc-RGD-BBN SPECT/CT with ^99m^Tc-3P4-RGD_2_ to assess the possible incremental value of ^99m^Tc-RGD-BBN SPECT/CT in breast cancer detection for the first time.

## Material and Methods

### Subjects

**Fig 1:**
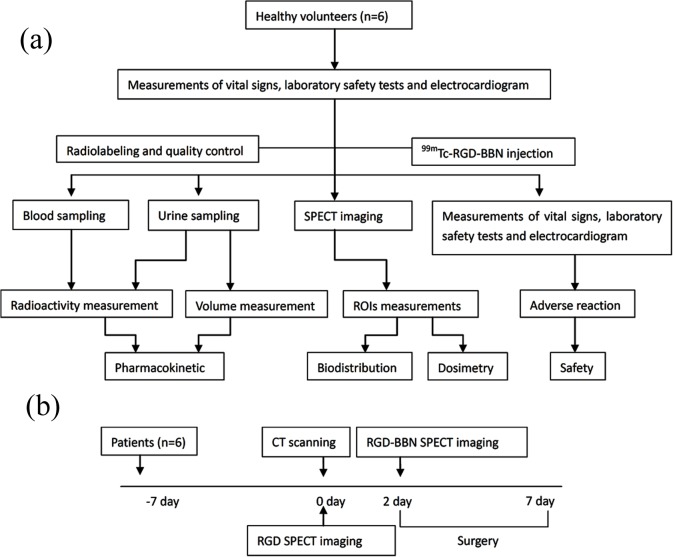
Flow chart of the study protocol for healthy volunteers (a) and patients (b), respectively.

6 patients (mean age 59 ± 10yr) with breast malignant tumor diagnosed by fine needle biopsy (FNAB) at least 7 days prior to surgery were included in the study. The healthy volunteers consisted of 3 males and 3 females aged between 25 and 51 years (Table 1). Physical examination and laboratory results from the last 6 months demonstrated no pathologic findings for all volunteers. Informed written consent to participate in the SMM studies was obtained from all subjects. This study was approved by the Ethics Committee of China-Japan Union Hospital of Jilin University. The flow chart of the study protocol for healthy volunteers and patients are shown in Fig 1.

**Table 1:** Demographic and Clinical Characteristics of Volunteers Investigated.

Volunteer No./Sex	Age (y)	Height (cm)	Weight (kg)	Body mass index (kg/m^2^)	Injected Activity (MBq)
1/M	51	175	73.5	24.00	1076.7
2/M	30	174	70.0	23.12	952.75
3/M	27	173	77.0	25.73	1061.9
4/F	25	155	41.0	17.07	1128.5
5/F	41	161	58.0	22.38	954.6
6/F	35	158	52.0	20.83	995.3
Mean±SD	34.83±9.81	166±8.99	61.92±13.99	22.19±2.99	1028.29±71.75

All patients were sent to the ^99m^Tc-3P4-RGD_2_ and ^99m^Tc-RGD-BBN SMM on an individual basis. The time interval between two imaging procedures was 48–54 hr. Finally, ^99m^Tc-RGD-BBN and ^99m^Tc-3P4-RGD_2_ SMM results were compared with each other. To be included, a patient had to be without any other history of breast disease. Exclusion criteria included pregnancy, lactation and a body weight greater than 80 kg.

A final diagnosis of the specimens obtained by the surgical procedure was made by histopathology. The most representative samples were submitted to immunohistochemical evaluation. For the resected lesions, the largest dimension of the tumor was considered the pathologic size. Results of SPECT/CT were compared with histological findings.

### ^99m^Tc-3P4-RGD_2_ and ^99m^Tc-RGD-BBN SPECT/CT

The 3P4-RGD_2_ and RGD-BBN were generously provided by the Medical Isotopes Research Center of Peking University in freeze-dried kits form. Na^99m^TcO_4_ was obtained from a commercial ^99^Mo/^99m^Tc generator (Beijing Atom High Tech Co., Ltd.). The kit for preparation of ^99m^Tc-3P4-RGD_2_ was formulated by containing, per millilitre, 20 μg of HYNIC-3P4-RGD_2_, 5 mg of TPPTS, 6.5 mg of tricine, 40 mg of mannitol, 38.5 mg of disodium succinate hexahydrate and 12.7 mg of succinic acid. Radiolabeling and quality control procedures for ^99m^Tc-3P4-RGD_2_ were performed as described previously ^4^. The kit for preparation of ^99m^Tc-RGD-BBN was formulated by containing, per millilitre, 20 μg of HYNIC-RGD-BBN, 6 mg of TPPTS, 10 mg of tricine, 40 mg of mannitol, 38.5 mg of disodium succinate hexahydrate and 12.7 mg of succinic acid [9]. Then 1 mL of Na^99m^TcO_4_ solution in saline was added to each kit vial followed by 30 min incubation at 100°C. The quality control was conducted by radioactive thin layer chromatography (ITLC), counting the high labeling yield of about 95%. Both ^99m^Tc-3P4-RGD_2_ and ^99m^Tc-RGD-BBN with a mean radioactivity of 299 ± 30 MBq and 293 ± 32 MBq were administered via single intravenous bolus injection followed by a 10 mL saline flush. ^99m^Tc-3P4-RGD_2_ and ^99m^Tc-RGD-BBN was performed with SPECT/CT at 1 h after intravenous injection of radiotracers respectively on separate days.

SPECT: Emission images were acquired using a dual-head, large field-of-view scintillation camera (Precedence, Philips Healthcare), equipped with a low-energy, high-resolution and parallel-hole collimator. In all healthy volunteers, planar images in both anterior and posterior views were acquired at 10 min, 30 min, 1 h, 2 h, 4 h, 8 h and 24 h post-injection and were stored digitally, using ^99m^Tc with a 20% energy window centered on 140 keV and 128×128 matrix. The velocity of scanning was 15 cm/min. SPECT images in all the patients were acquired over 360° (180°per head) in supine position with raised arms during the imaging. Imaging with both radiotracers was performed using 6°angular steps in a 20 s time frame, with a 64×64 matrix size. Distance between the breast and detector was minimized. The position of the patient was recorded in detail and kept consistent to the greatest extent in the second SPECT scan to make sure that the two different SPECT images could be fused to the CT images.

CT images from neck to abdomen were acquired sequentially in a non-dedicated 3rd-generation scanner installed in the SPECT camera gantry, with a 10 mm slice thickness, a maximum current of 2.5 mA and a 140 kV potential. The patients just had one CT scan since ^99m^Tc-3P4-RGD_2_ and ^99m^Tc-RGD-BBN SPECT were undertaken on the same device.

All images were interpreted qualitatively by three experienced nuclear medicine radiologists who were unaware of the clinical history and other test results of all patients. Visual analysis was performed on a per-lesion basis and in a blinded fashion. A 3-point scale system was adopted to describe the uptake degree for breast lesions. The rules for classification were as follows: grade 1, no abnormal increased uptake (not higher than contralateral or peripheral normal breast tissue); grade 2, mildly increased uptake (lower than mediastinum or equivalent to mediastinum); grade 3, definite focal increased uptake (higher than mediastinum). Homogeneous uptake in both breasts was classified as grade 1. SMM was considered positive for malignancy if the visual score was ≥2. There was no disagreement between three radiologists in our study.

### Immunohistochemistry of α_v_β_3_ and GRPR expression

The immunohistochemistry of α_v_β_3_ and GRPR expression of the breast tissue sample was performed as described previously with some modifications [4,10]. The tissues were snap-frozen, sectioned (3 μm) and fixed with ice-cold acetone, rinsed with PBS and blocked with 10% goat serum for 30 min at room temperature. The slices were incubated with goat anti-GRPR antibody (1:100; Santa Cruz Biotechnology, CA), humanized antihuman integrin α_v_β_3_ antibody Abegrin (20 μg/mL) (1:100; BD Biosciences, CA) for 1h at room temperature. Immunohistochemical staining was scored according to intensity and distribution in the following way: 1. no staining, 2. weak staining, 3. strong staining; 1. no cells stained, 2. less than 10% cells stained, 3. 10–50% cells stained, 4. 50–90% cells stained, 5. all cells stained. For the purposes of data presentation, tumors were considered positive if the sum score of intensity and distribution was > 6—that is, strong staining in at least 10% of cells or weak staining in over half of the tumor cell population [11].

### Blood and urine sampling

The ^99m^Tc-RGD-BBN with a range of 786.7 ± 55.8 MBq (19.1–24.2 mCi) was injected into the antecubital vein of all healthy volunteers with a rapid bolus, followed by a 10 mL saline flush. Measurements of vital signs (body temperature, systolic and diastolic blood pressure and pulse rate), laboratory safety tests (renal and liver function chemistry, hematology, and blood coagulation parameters) and 12-lead electrocardiogram were recorded before and after tracer injection.

1.5 mL venous blood sampling was collected via an indwelling catheter throughout the imaging period specifically at 1, 3, 5, 10, 15, 30, 60 and 120 minutes post-injection. Samples were weighed and counted in a γ-counter (Wallac 1470–002,Perkin Elmer,Finland). Decay corrected time-activity curve was expressed as percentage of injected dose per gram (%ID/g).

A urine sample was collected at the following hourly intervals after tracer injection: 0 to 2, 2 to 4, 4 to 8, 8 to 12 and 12 to 24 hours. Samples had been weighing the volume. Samples’ radioactivity was measured with the gamma counter.

### Biodistribution and dosimetry estimation

Visual analysis was applied to determine the integral biodistribution of the tracer and transient and intersubject stability. For each subject, regions of interest (ROIs) were delineated over the identified organs including: lung, heart, liver, kidneys, spleen, intestine, urinary bladder and a background region near the body on the anterior image. The mirror ROIs were applied to the posterior images of each organ. The mean counts of each organ on planar images were measured. The results were expressed as percentage of initial injected activity after decay-correction.

Fitted residence time functions were plotted and multiplied by the exponential decay functions for ^99m^Tc. These functions were then integrated analytically to determine the area under the curve (AUC) to yield the residence time of each organ. Then, these residence times were input in OLINDA/EXM 1.0 software (Vanderbilt, University, Nashville, TN) to calculate equivalent organ doses and the effective dose (ED) based on the 70-kg reference adult phantom in International Commission on Radiological Protection (ICRP) publication 60 [12].

### Statistical analysis

By using SPSS 13.0 statistical analysis software, data are expressed as mean ± SD. A two-tailed Student’s *t* test for was employed to assess the statistical differences in T/N ratios. A *P* value of <0.05 was considered statistically significant.

## Results

No clinically significant abnormalities or abnormal clinical chemistry were reported in any of the patients during the study. The results of measurements of vital signs (body temperature, systolic and diastolic blood pressure and pulse rate), laboratory safety tests (renal and liver function chemistry, hematology, and blood coagulation parameters) and 12-lead electrocardiogram were normal before and after the study.

### The pharmacokinetic results

**Fig 2:**
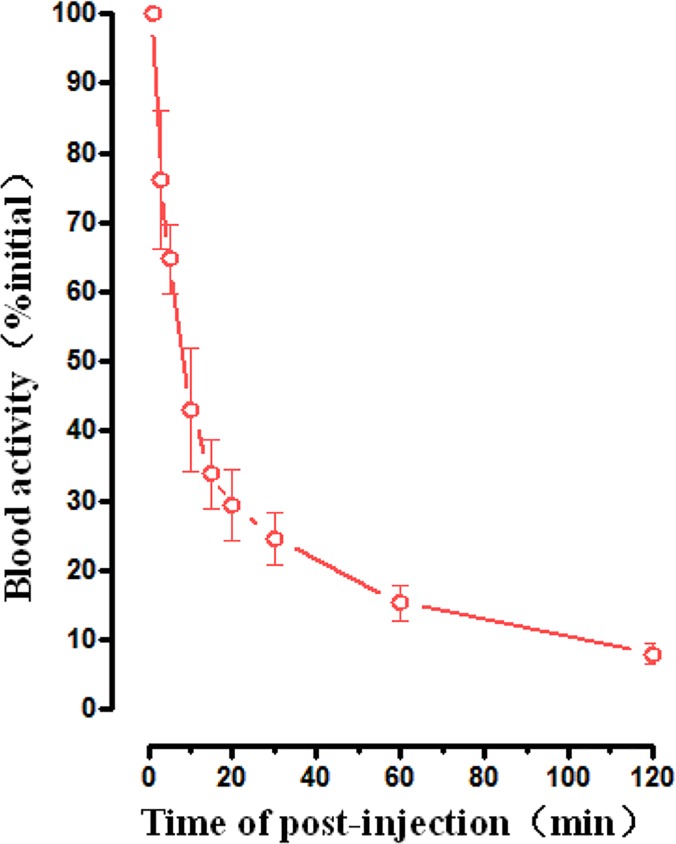
Averaged time-activity curve of ^99m^Tc-RGD-BBN in blood for all healthy volunteers. Error bars indicate standard deviations.

Fig 2 gives the time-activity curve reporting the blood clearance over the first 120 min respectively. The data showed there was a very sharp decline of radioactivity in the blood. Blood activity was 43.05%, 24.60% and 15.33% of the initial dosage at 10, 30 and 60 min respectively after injection.

**Fig 3:**
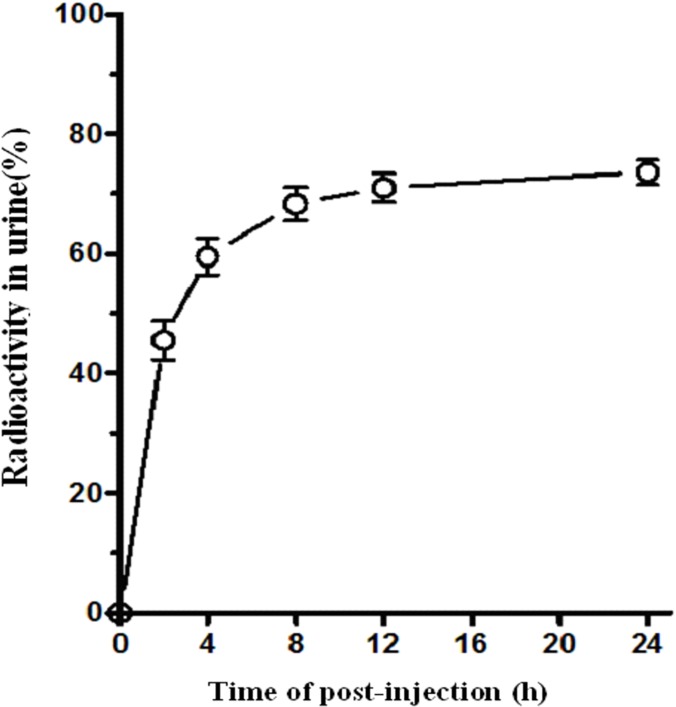
Averaged time-activity curve of ^99m^Tc-RGD-BBN in urine for all healthy volunteers. Error bars indicate standard deviations.

The concentration of radioactivity in the urine was shown in Fig 3. The radioactivity in the urine keeps increasing with a total cumulative recovery of (73.56 ± 2.04) % of the original dose at 24h.

### Biodistribution of ^99m^Tc-RGD-BBN in normal subjects

**Fig 4:**
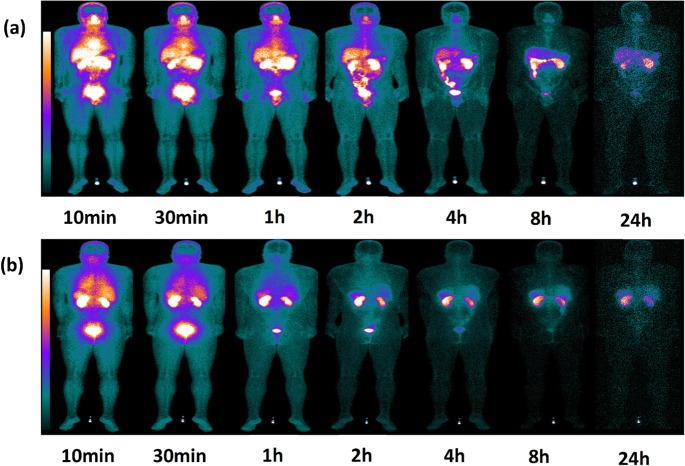
Series of planar whole-body images of a representative subject showing the distribution of ^99m^Tc-RGD-BBN between 10 min and 24 h post-injection. (a) Anterior planar whole-body images. (b) Posterior planar whole-body images.

Fig 4 shows a representation of a coronal section from whole-body SPECT and the distribution of ^99m^Tc-RGD-BBN at 10 min, 30 min, 1 h, 2 h, 4 h, 8 h and 24 h post-injection. The predominant uptake was seen in the bladder, indicating a renal-urinary excretion of the tracer. The kidneys and liver also showed moderate uptake. Apparent tracer uptake was observed in the nasal cavity and salivary glands in the early time points and almost undetectable at 24 hr after injection.

**Fig 5:**
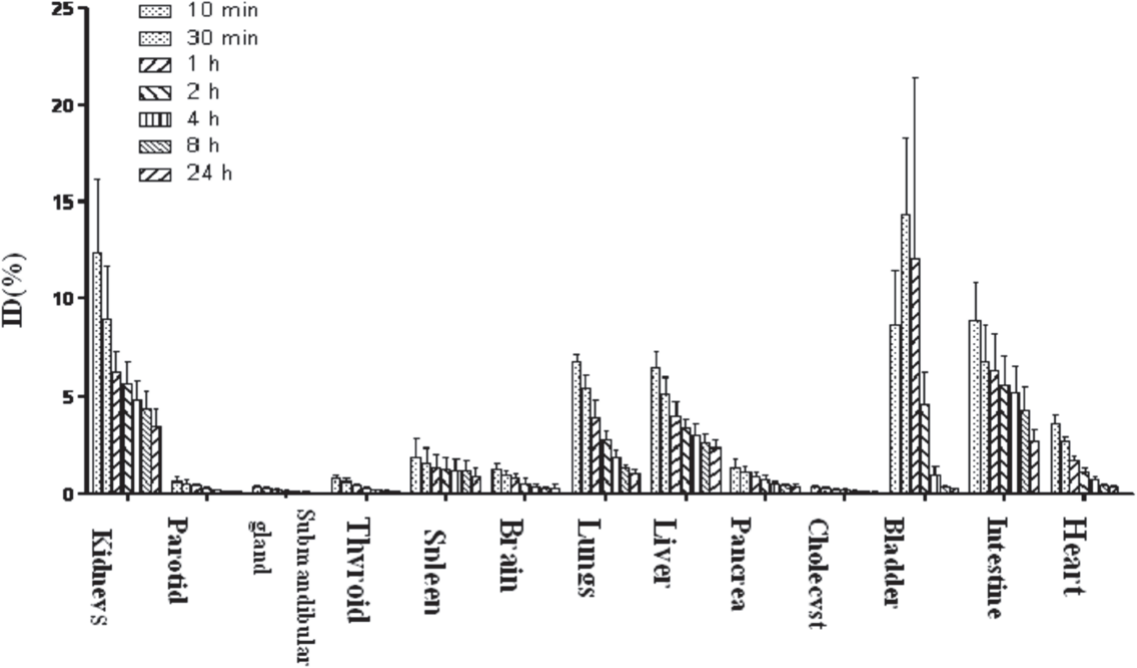
The quantified analysis of ^99m^Tc-RGD-BBN in major organs of healthy volunteers calculated from the whole-body images obtained at 10 min, 30 min, 1 h, 2 h, 4 h, 8 h and 24 h after administration.

By measuring ROIs drawn on both anterior and posterior images, the quantitative tracer uptakes in major organs were presented in Fig 5. It shows the highest activity in visceral organs was found in the bladder which ascended from 8.71 ± 2.33% ID/organ (10 min p.i.) to 12.05 ± 8.40% ID/organ (60 min p.i.). It was followed by the kidneys and the liver which declined over time (10 min p.i. to 60 min p.i., 12.40 ± 3.37% to 6.24 ± 1.11% ID/organ, 6.47 ± 0.67% to 4.04 ± 0.63% ID/organ, respectively). Low activities were remained in the spleen and heart till 60 min after injection (1.35 ± 0.49% and 1.17 ± 0.24% ID/organ, respectively).

### Dosimetry

A summary of dosimetric parameters for various organs and whole body is given in Table 2. The mean effective dose equivalent for the whole body was 2.17E-03 mSv/MBq.

**Table 2:** Dosimetric data of ^99m^Tc-RGD-BBN in all healthy volunteers (n = 6).

Target Organ	Dosimetric data(×10^–3^ mSv/MBq)		
	Male(n = 3)	Female(n = 3)	Total(n = 6)
Adrenal glands	3.53±0.40	4.16±0.92	3.85±0.66
Brain	1.11±0.19	0.86±0.26	0.99±0.22
Breasts	1.08±0.12	1.17±0.27	1.12±0.18
Gallbladder wall	5.09±0.79	5.22±1.13	5.15±0.80
Lower region of colon	19.40±2.18	27.37±7.33	23.38±5.95
Small intestine	3.08±0.28	3.86±0.76	3.47±0.61
Stomach wall	2.66±0.21	2.93±0.56	2.80±0.37
Upper colon	2.50±0.24	3.04±0.64	2.77±0.48
Heart wall	3.57±0.49	3.77±0.54	3.67±0.43
Kidneys	23.60±4.24	27.50±7.78	25.55±5.48
Liver	5.06±0.51	5.45±1.29	5.26±0.82
Lungs	3.96±0.41	4.48±1.05	4.22±0.70
Muscle	1.65±0.14	1.88±0.40	1.77±0.27
Ovaries	-	4.54±0.95	4.54±0.95
Pancreas	9.68±3.03	11.43±1.36	10.56±2.11
Red marrow	1.88±0.20	2.19±0.47	2.03±0.33
Osteogenic cells	3.80±0.42	4.28±1.01	4.04±0.68
Skin	0.91±0.10	0.99±0.23	0.95±0.15
Spleen	14.93±1.96	9.59±4.21	12.26±3.78
Testis	1.48±0.05	-	1.48±0.05
Thymus	1.60±0.20	1.71±0.37	1.66±0.25
Thyroid gland	14.16±4.71	14.57±1.17	14.37±2.81
Urinary bladder wall	12.25±2.99	16.50±5.89	14.38±4.37
Uterus	-	4.14±0.77	4.14±0.77
Whole body	2.02±0.19	2.31±0.49	2.17±0.34

### Scintimammography

**Fig 6:**
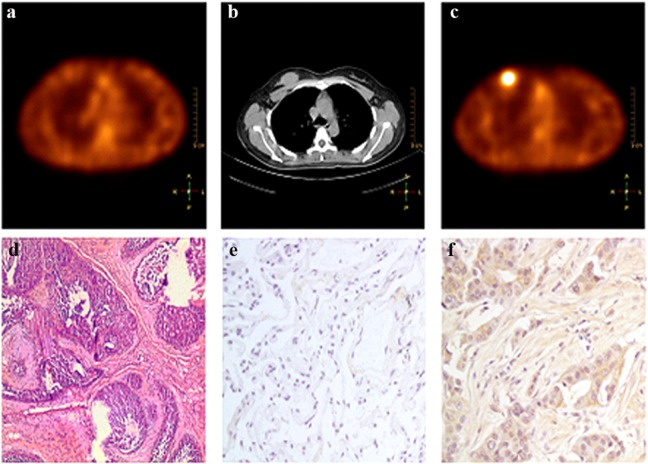
A 51-y-old patient with a breast malignant tumor in the right side as Patient 1 in Table 3; (a) ^99m^Tc-3P4-RGD_2_ SMM demonstrates no tracer uptake observed in the lesion. (b) CT scan demonstrates a mass in the right breast. (c) ^99m^Tc-RGD-BN SMM demonstrates high uptake observed in the lesion. (d) Histopathology staining indicated a ductal carcinoma in situ. ×40 (e) Immunohistochemistry demonstrates barely α_v_β_3_ expression in tumor vessels and tumor cells. ×400. (f) Immunohistochemistry demonstrates intense GRPR expression in tumor vessels and tumor cells. ×400.

All underwent surgery within 1 wk. A total of 6 palpable malignant lesions in 6 patients were described in the standard of truth including 4 invasive ductal carcinomas (IDC) and 2 ductal carcinoma in situ (DCIS) (Table 3). All of them were detected by the prescribed ^99m^Tc-RGD-BBN SPECT/CT imaging with clear uptake. All malignant lesions were also detected using ^99m^Tc-3P4-RGD_2_ SPECT/CT and the result is five malignant lesions with clear uptake and the other one with barely an uptake (Fig 6).

**Table 3:** Characteristics of all patients.

Patients	Pathology	Location	Lesion size(cm)	Visual uptake grading	RGD (T/N Ratio)	Visual uptake grading	RGD-BBN (T/N Ratio)	Immunohistochemistry	
				RGD		RGD-BBN		α_v_β_3_	GRPR
1	DCIS	R	2.3	1	1.04	3	3.31	-	+
2	DCIS	L	2.0	3	3.09	3	2.72	+	+
3	IDC	L	5.1	3	3.12	3	3.06	+	+
4	IDC	R	2.1	3	2.56	3	2.45	+	-
5	IDC	L	5.8	3	4.43	3	4.17	+	+
6	IDC	L	6.3	3	3.12	3	3.03	+	+

SPECT/CT with ^99m^Tc-RGD-BBN and ^99m^Tc-RGD: 3-grade scale, where grade 1 = no abnormal increased uptake; grade 2 = mildly increased or heterogeneous uptake; grade 3 = definite focal increased uptake. IDC = infiltrative ductal carcinoma; DCIS = ductal carcinoma in situ.

### Immunohistochemistry of α_v_β_3_ expression

For 6 malignant samples, 4 cases were found with dual α_v_β_3_ and GRPR expression, 1 case with only GRPR positive expression (integrin α_v_β_3_ negative) (Fig 5) and 1 case with only integrin α_v_β_3_ positive expression (GRPR negative) (Table 3). For the 4 cases with dual α_v_β_3_ and GRPR expression, there was no significant statistical difference in the T/N ratios between ^99m^Tc-RGD-BBN and ^99m^Tc-3P4-RGD_2_ imaging (t = 2.6681; *P* >0.05).

## Discussion

The SMM is less widespread than XMM and ultrasound US, but this does not mean that it has to remain in the background, as it is of great diagnostic value in breast tumor, especially after the emergence of SPECT/CT and the constant development of new tracers offers great potential for SMM. The combination of SMM and CT implies a huge progress due to its high diagnostic performance and ability to detect tumor pathological conditions. As non-invasive and sensitive imaging methods they have been widely used for diagnosing diseases in the clinic [13].

The emergence of various probes has become an indispensable factor for SMM. In previous studies, several one-target based radiotracers have been developed, such as a series of arginine-glycine-aspartic acid (RGD) containing peptides and radiolabeled BBN analogue which can specifically bind to integrin α_v_β_3_ and GRPR respectively[14–19]. Some have been successfully applied to human clinical trials [20]. But monomeric RGD or BBN based radiotracers have some drawbacks for tumor imaging. First, not all tumors highly express cell-surface targets during progression of tumor initiation, growth, invasion and relapse. For instance, GRPR expression is greatly reduced during the dedifferentiation of prostate cancer cells from androgen-controlled to androgen-independent transformation [21]. Second, the relatively feeble binding affinity and unsatisfactory in vivo pharmacokinetics of the monomeric radiotracers may lead to rapid dissociation from the tumor targets and the fuzzy imaging. For these reasons, dual targeted peptide provides a new avenue for SMM. It could be with more tumor binding affinity and could identify tumor in its various pathological process. Recently, a dual integrin α_v_β_3_ and GRPR targeted peptide ^99m^Tc-RGD-BBN was developed and investigated. Data from previous studies in xenografts mouse models showed ^99m^Tc-RGD-BBN has excellent behaviors in vivo and in vitro and exhibited specific tumor imaging with high contrast to the contralateral background [8,9]. Based on these favorable study results and our continuous interest in dual-targeted molecular probes for cancer imaging, we tentatively applied this new radiotracer in 6 healthy volunteers and 6 patients with breast cancer for the first time.

No clinically significant abnormalities or abnormal clinical chemistry were reported in any of the patients during our study. This showed that ^99m^Tc-RGD-BBN had good security and stability for human use. The pharmacokinetic results were also satisfactory. There was a very sharp decline of radioactivity in the circulation and the radioactivity in the urine kept increasing with a total cumulative recovery of (73.56 ± 2.04) % of original dose at 24 h. The biodistribution of ^99m^Tc-RGD-BBN in normal subjects indicated a renal-urinary excretion of the tracer.

The effective radiation dose to the body of ^99m^Tc-3P4-RGD_2_ and ^99m^Tc-RGD-BBN were 1.15 ± 0.13 mSv and 0.65 ± 0.07 mSv respectively ^9^. According to the 2007 Recommendations of the International Commission on Radiological Protection (ICRP) [22–24], radiation equivalent dose which occupational workers’ vital organs were exposed to should be less than 500mSv and radiosensitive organs should be less than 150mSv. In our study, the mean absorbed dose equivalent for the whole body was 2.02E-03 mSv / MBq in healthy males and 2.31E-03 mSv / MBq in healthy females. Compared with other clinical markers of ^99m^Tc, ^99m^Tc-RGD-BBN is significantly lower than the conventional nuclear medical imaging agent, such as ^99m^Tc—MDP bone check (5.92 mSv) and ^99m^Tc—MIBI heart examination (7.05 mSv) [25].

All of the 6 malignant lesions were clearly detected by ^99m^Tc-RGD-BBN SPECT/CT imaging with intense uptake, which is consistent with the recent report of ^18^F, ^64^Cu and ^68^Ga labeled RGD-BBN PET imaging [8]. ^99m^Tc-3P4-RGD_2_ SPECT/CT imaging showed five lesions with clear uptake and the other one with barely uptake. This case was found with only GRPR positive expression (integrin α_v_β_3_ negative), which could be the reason for the different imaging result by ^99m^Tc-3P4-RGD_2_ and ^99m^Tc-RGD-BBN. The advantage of dual integrin α_v_β_3_ and GRPR targeted peptide is obvious when only one receptor type is overexpressed. For example, in the lesion of patient 1, which expressed GRPR but no integrin α_v_β_3_, ^99m^Tc-3P4-RGD_2_ imaging was unable to detect the tumor because it only recognized integrin α_v_β_3_. In contrast, ^99m^Tc-RGD-BBN imaging had a clear tumor uptake due to the function of GRPR. Therefore, ^99m^Tc-RGD-BBN may have the potential to make up for the deficiency of ^99m^Tc-3P4-RGD_2_ in the detection of breast malignant tumors with integrin α_v_β_3_ negative expression but GRPR positive expression. There was one case with only integrin α_v_β_3_ positive expression (GRPR negative), the T/N ratio of ^99m^Tc-3P4-RGD_2_ imaging was higher than that of ^99m^Tc-RGD-BBN imaging (2.56 vs. 2.45), which might have resulted from the improved in tumor affinity and pharmacokinetic of dimeric RGD peptide over monomeric RGD. The immunohistochemistry revealed 4 cases with both integrin αvβ3 and GRPR positive expression, the T/N ratio of ^99m^Tc-3P4-RGD_2_ imaging was higher than that of ^99m^Tc-RGD-BBN imaging but there was no significant statistical difference (*P* >0.05). The reasons for this may be as follows: ^99m^Tc-3P4-RGD_2_ is a well-designed, dimeric RGD peptide which can bind to two integrin αvβ3 motifs simultaneously. Some studies found that ^99m^Tc-RGD-BBN is impossible to bind to both GRPR and integrin simultaneously in vivo since the glutamate linker between the RGD and BBN motifs is not long enough. The total number of binding sites (the sum of GRPR and integrin) for ^99m^Tc-RGD-BBN would significantly increase as compared to the monomeric counterparts in the dual-receptor positive tumor models, which would lead to improve in vivo tumor targeting efficacy. Since the expression of cell-surface receptors by cancer cells can be heterogeneous and inhomogeneous, it is very hard to distinguish the expression level of each receptor individually when using dual-targeted molecular probes such as ^99m^Tc-RGD-BBN. The uptake of ^99m^Tc-3P4-RGD_2_ by tumor with both integrin α_v_β_3_ and GRPR positive expression may be higher than ^99m^Tc-RGD-BBN if more integrin α_v_β_3_ are expressed. However, due to the small sample size of this study, there was no significant statistical difference in T/N ratios between ^99m^Tc-3P4-RGD_2_ imaging and ^99m^Tc-RGD-BBN imaging.

It is well-known that a realistic strategy for the reduction of breast cancer mortality rates and timely treatment is to detect the disease as early as possible. The most common screening method for early breast cancer is mammography. Our previous research of ^99m^Tc-3P4-RGD_2_ imaging has shown promise as an additional tool to mammography to avoid unnecessary biopsies [26]. A breast tumor is always estrogen dependent (ER+) or estrogen independent (ER-) [27]. Some ER+ cells are with positive GRPR expression but with negative integrin αvβ3 expression. For ER- cells, this often is not the case. Therefore, the dual integrin αvβ3 and GRPR targeting probe ^99m^Tc-RGD-BBN makes it possible to detect tumors with either integrin αvβ3 or GRPR expression patterns [9]. The promising imaging results of ^99m^Tc-RGD-BBN in patients with breast cancer may give rise to the possibility of extending applications in breast cancer screening to avoid unnecessary biopsies.

In this study, our findings are of a preliminary nature and need to be further corroborated. It is necessary to investigate the effects of linkers of different lengths, solubility, lipophilicity, and flexibility on the in vitro and in vivo behaviors of the dual integrin α_v_β_3_ and GRPR targeted peptide to make it possible to bind to both receptors simultaneously, which may result in enhanced targeting efficacy and higher uptake of ^99m^Tc-RGD-BBN by the tumor. This may help to provide new idea in the development of tumor targeted therapy. The design of heteromultimeric tracers that recognize other tumor targets is also worth further investigation for tumor-targeted imaging and therapy.

There are several limitations to this study that call for further discussion. First, as a combined probe, the sensitivity of ^99m^Tc-RGD-BBN SPECT/CT in detecting breast malignant tumor should be improved, but the specificity may be also affected. In our study, there was no false positive or false negative case by ^99m^Tc-RGD-BBN SPECT/CT imaging. Such a high accuracy might be related to the small numbers of patients included in this study and all of them were with breast malignant tumor, which was chosen with a subjective wish. Further research is needed to include a larger patient population. Second, although the immunohistochemistry of integrin α_v_β_3_ and GRPR expression were conducted in this study, quantification and correlation with tracer uptake was not performed due to the heterogeneous and inhomogeneous expression of cell-surface receptors by cancer cells.

### Conclusions

The dual integrin α_v_β_3_ and GRPR targeting ^99m^Tc-RGD-BBN showed an excellent ability to detect breast cancer without clinically safety problems. ^99m^Tc-RGD-BBN may have the potential to make up for the deficiency of ^99m^Tc-3P4-RGD_2_ in the detection of breast cancer with only GRPR positive expression (integrin α_v_β_3_ negative). The preliminary application of ^99m^Tc-RGD-BBN has demonstrated its powerful potential in breast cancer diagnosis and therapy.
